# Cardiac function and intracellular Ca^2+^ handling proteins are not impaired by high-saturated-fat diet-induced obesity

**DOI:** 10.1590/1414-431X20198085

**Published:** 2019-05-27

**Authors:** A.F. Deus, D.F. Vileigas, D.C.T. Silva, L.C. Tomasi, D.H.S. Campos, K. Okoshi, C.R. Padovani, A.C. Cicogna

**Affiliations:** 1 Departamento de Clínica Médica, Faculdade de Medicina de Botucatu, Universidade Estadual Paulista, Botucatu, SP, Brasil; 2 Departamento de Bioestatística, Instituto de Biociências de Botucatu, Universidade Estadual Paulista, Botucatu, SP, Brasil

**Keywords:** Obesity, Saturated fatty acids, High-fat diet, Cardiac function, Calcium handling proteins, Rat

## Abstract

Obesity is often associated with changes in cardiac function; however, the mechanisms responsible for functional abnormalities have not yet been fully clarified. Considering the lack of information regarding high-saturated-fat diet-induced obesity, heart function, and the proteins involved in myocardial calcium (Ca^2+^) handling, the aim of this study was to test the hypothesis that this dietary model of obesity leads to cardiac dysfunction resulting from alterations in the regulatory proteins of intracellular Ca^2+^ homeostasis. Male Wistar rats were distributed into two groups: control (C, n=18; standard diet) and obese (Ob, n=19; high-saturated-fat diet), which were fed for 33 weeks. Cardiac structure and function were evaluated using echocardiographic and isolated papillary muscle analyses. Myocardial protein expressions of sarcoplasmic reticulum Ca^2+^-ATPase, phospholamban (PLB), PLB serine-16 phosphorylation, PLB threonine-17 phosphorylation, ryanodine receptor, calsequestrin, Na^+^/Ca^2+^ exchanger, and L-type Ca^2+^ channel were assessed by western blot. Obese rats presented 104% increase in the adiposity index (C: 4.5±1.4 *vs* Ob: 9.2±1.5%) and obesity-related comorbidities compared to control rats. The left atrium diameter (C: 5.0±0.4 *vs* Ob: 5.5±0.5 mm) and posterior wall shortening velocity (C: 36.7±3.4 *vs* Ob: 41.8±3.8 mm/s) were higher in the obese group than in the control. The papillary muscle function was similar between the groups at baseline and after inotropic and lusitropic maneuvers. Obesity did not lead to changes in myocardial Ca^2+^ handling proteins expression. In conclusion, the hypothesis was not confirmed, since the high-saturated-fat diet-induced obese rats did not present cardiac dysfunction or impaired intracellular Ca^2+^ handling proteins.

## Introduction

The incidence and prevalence of obesity has increased worldwide, representing a pandemic and, consequently, a public health problem (1). Excess adiposity is strongly associated with increased mortality risk, reduction in life expectancy, and the development of major risk factors for numerous co-morbidities such as type II diabetes mellitus, hyperlipidemia, and cardiovascular diseases (2,3).

Current experimental studies with diet-induced obesity models focus not only on the amount of fat used but also on the source, resulting in diets with a predominance of saturated or unsaturated fatty acids, since they are substantially associated with different outcomes (4). In addition, research studies confirm that dietary saturated fatty acids are positively associated with increased ventricular remodeling, cardiac hypertrophy, and mitochondrial and contractile dysfunction (5–8).

The mechanisms responsible for functional abnormalities in obesity have not yet been fully clarified. Many factors have been presented as responsible for the cardiac changes in obesity, including intracellular calcium (Ca^2+^) handling (9–12). The intracellular Ca^2+^-cycling proteins located in the sarcolemma and sarcoplasmic reticulum (SR), such as the L-type Ca^2+^ channel, SR Ca^2+^-ATPase (SERCA2a), phospholamban (PLB), calsequestrin (CSQ), ryanodine receptor (RYR), and Na^+^/Ca^2+^ exchanger (NCX), are important in the regulation of myocardial contraction and relaxation by controlling calcium transient homeostasis (12,13). Thus, changes in proteins involved in coordinating Ca^2+^ movement may contribute to contractile dysfunction.

Most studies found in the literature that assessed cardiac function and calcium handling-related proteins in obesity models used high-fat diets with a predominance of unsaturated fatty acids (9,14–18). However, Cheng et al. (19), using a high-fat diet rich in saturated fatty acids for 8 weeks, showed that only pPLB Thr^17^/PLB ratio was downregulated, without altering the cardiac function and the protein levels of SERCA2a, PLB, pPLB Ser^16^, as well as the ratios of SERCA2a and pPLB Ser^16^ to total PLB. There is no study that evaluated all proteins related to myocardial Ca^2+^ handling in long-term obesity induced by high-fat diet with a predominance of saturated fatty acids. Therefore, the aim of this study was to test the hypothesis that this dietary model of obesity causes cardiac dysfunction resulting from alterations in the regulatory proteins of intracellular calcium homeostasis. Taken together, the results will contribute to the understanding of the mechanisms underlying the participation of this pathway in heart function during a prolonged period of obesity.

## Material and Methods

### Animals and experimental protocol

Sixty-day-old male Wistar rats were obtained from Anilab Animais de Laboratório Criação e Comércio (Paulínia, Brazil). After 7 days of acclimatization, the rats were randomized into two groups: control (C, n=18) and obese (Ob, n=19), which were fed a standard diet (SDiet) and a high-saturated-fat diet (HSFDiet), respectively, for 33 weeks. All rats had free access to food and water. The animals were housed in individual cages with controlled temperature (24±2°C), humidity (55±5%), and light (12-h light/dark cycle). The experimental protocol was approved by the Ethics Committee on Animal Experimentation of the Botucatu Medical School, UNESP (protocol no. 994/2012). All procedures were in accordance with the “Guide for the Care and Use of Laboratory Animals, 8th Edition” published by the U.S. National Research Council (20).

### Diets

The diets were developed at the Experimental Research Unit (UNIPEX) of the Botucatu Medical School in partnership with Biotron Zootécnica® (Brazil), based on a dietary model previously used by the group (21). The following ingredients were used to formulate both diets: corn bran, soybean hulls and bran, dextrin, salt, vitamin and mineral complex, palm kernel oil, and soybean oil. The SDiet contained 31.0% of its kcal from protein, 51.6% from carbohydrates, and 17.4% from fat; the HSFDiet values were 18.7% from proteins, 41.6% from carbohydrates, and 39.7% from fat. The HSFDiet was calorically richer (HSFDiet=3.85 kcal/g *vs* SDiet=3.10 kcal/g) due to higher energy from fat. The content of saturated/unsaturated fatty acids was 61.5/38.5% in the SDiet and 64.8/35.2% in the HSFDiet.

### Nutritional profile

The nutritional profile was evaluated according to the following parameters: food and calorie intake, feed efficiency, body weight, body fat, and adiposity index. Food intake and body weight of the animals were measured weekly. Calorie consumption was determined by multiplying the energy value of each diet (g×kcal) by the weekly food intake. To analyze the capacity of converting consumed food energy into body weight, feed efficiency was calculated by dividing the total body weight gain (g) by the total energy intake (kcal). The adipose tissue fat pads were dissected and weighed after the animals were anesthetized (50 mg/kg ketamine; 1 mg/kg xylazine; intraperitoneal injection [*ip*]; Sespo Indústria e Comércio Ltda - Divisão Vetbrands, Brazil) and decapitated. Total body fat was determined by the sum of the epididymal, retroperitoneal, and visceral fat pads. The adiposity index was calculated by total body fat divided by the final body weight and multiplied by 100.

### Obesity-related comorbidities

#### Systolic blood pressure (SBP)

At the end of the experimental protocol, one week before euthanasia, SBP was measured in conscious rats using the non-invasive tail-cuff method with an electro-sphygmomanometer, Narco Bio-System (International Biomedical, USA). The animals were warmed in a wooden box between 38 and 40°C, the heat was generated by two incandescent lamps for 4 min to cause vasodilation of the tail artery. Then, they were transferred to an iron cylindrical support that was specially designed to allow total exposure of the tail. After this process, a sensor coupled to the electro-sphygmomanometer was placed in the proximal region of the tail (22). The arterial pulsations were recorded in a computerized data acquisition system (Biopac Systems Inc., USA). On average, two readings were recorded for each measurement.

#### Metabolic and hormonal profile

The glucose tolerance test was performed one week before euthanasia. The animals were fasted for 6 hours and blood samples from the tail tip were collected at baseline and after intraperitoneal administration of 30% glucose solution, equivalent to 2.0 g/kg body weight. The samples were analyzed using a handheld glucometer (Accu-Chek Go Kit; Roche Diagnóstica Brasil Ltda, Brazil) at baseline (0 min) and after 15, 30, 60, 90, and 120 min of glucose injection. Glucose tolerance was assessed by the area under the curve (AUC). At the end of the experimental protocol, the animals were fasted for 12 h, anesthetized (50 mg/kg ketamine; 1 mg/kg xylazine; *ip*; Sespo Indústria e Comércio Ltda - Divisão Vetbrands), and euthanized by decapitation. Blood samples were collected, and the serum was separated by centrifugation at 1,620 *g* for 10 min at 4°C and stored at –80°C for later analysis. Triacylglycerol, total cholesterol, high-density lipoprotein (HDL), and low-density lipoprotein (LDL) concentrations were determined using specific kits (BIOCLIN®, Brazil) in an automated chemical analyzer BS-200 (Mindray, China). The non-esterified fatty acids (NEFA) levels were evaluated using a colorimetric kit (WAKO Pure Chemical Industries Ltd, Japan). Leptin and insulin levels were analyzed by the enzyme-linked immunosorbent assay (ELISA) method (EMD Millipore Corporation, USA). For glucose analysis, the animals were exposed to fasting and anesthesia, as described above, and blood samples were collected from the tail tip; the glucose levels were assessed using a handheld glucometer. The homeostatic model assessment of insulin resistance (HOMA-IR) was used as an insulin resistance index, calculated according to the formula: HOMA-IR = [fasting glucose (mmol/L) × fasting insulin (µU/mL)] / 22.5 (23).

### Cardiac remodeling

#### Macroscopic analysis postmortem

Cardiac remodeling was evaluated by postmortem analysis of the following parameters: atria (A) and left (LV) and right (RV) ventricles weights, and their ratio with tibia length.

#### Echocardiographic evaluation

The echocardiographic study was performed one week before euthanasia using a commercially available echocardiography machine (General Electric Medical Systems, Vivid S6, Israel) equipped with a 5–11.5 MHz electronic transducer, as previously described (10 [Bibr B11],^24^). Briefly, the rats were anesthetized via *ip* injection of a mixture of ketamine (50 mg/kg) and xylazine (1 mg/kg). A two-dimensional parasternal short-axis view of the LV was obtained at the level of the papillary muscles. M-mode tracings were obtained from short-axis views of the LV at or just below the tip of the mitral valve leaflets, and at the level of the aortic valve and left atrium. M-mode images of the LV were printed on a black-and-white thermal printer (Sony UP-890MD, Japan) at a sweep speed of 100 mm/s. All cardiac structures were manually measured with a caliper by the same researcher, according to the method of the American Society of Echocardiography (25). Measurements were recorded as the mean of at least five consecutive cardiac cycles. The following LV structural parameters were evaluated: LV diastolic diameter (LVDD), LV diastolic posterior wall thickness (LVDPWT), LV relative wall thickness (LVRWT = 2 × LVDPWT / LVDD), and left atrium (LA) and aorta (AO) diameters. LV function was evaluated by the following parameters: heart rate (HR), endocardial fractional shortening (FS), posterior wall shortening velocity (PWSV), early and late diastolic mitral inflow velocities (E and A waves), and E/A ratio.

#### Isolated papillary muscle function

Cardiac contractile performance was evaluated by studying the isolated papillary muscle from the LV as previously described (10,16). The following mechanical parameters were measured from isometric contraction: maximum developed tension (DT; g/mm^2^), resting tension (RT; g/mm^2^), and peak of positive (+dT/dt; g/mm^2^/s) and negative (−dT/dt; g/mm^2^/s) tension derivatives. The mechanical behavior of the papillary muscle was evaluated under baseline conditions at 2.5 mM Ca^2+^ and after inotropic and lusitropic maneuvers: length-tension relationship, increases in extracellular Ca^2+^ concentrations (to test their effects on myofilament machinery), and post-rest contraction (PRC), mainly related to SR storage and release capacity. The length-tension relationships were characterized by determining the RT (myocardial stiffness) and DT (Frank-Starling curve) at baseline (100% L_max_), and 98, 96, 94, and 92% of optimum length. RT-length and DT-length curves were plotted using regression analysis: log(RT) = −51.1118 + 25.5425 log(L_max_) for the C group; log(RT) = −58.1992 + 29.1455 log(L_max_) for the Ob group; DT = 29.444 − 2349.613 / L_max_ for the C group; DT = 29.232 − 2285.702 / L_max_ for the Ob group. Inotropic responses were recorded 5 min after the addition of each dose of extracellular Ca^2+^ (0.5, 1.0, 1.5, 2.0, and 2.5 mM) to the bathing solution. PRC was studied at an extracellular Ca^2+^ concentration of 0.5 mM, the stimulus was paused for 10, 30, and 60 s before restarting the stimulation. During rest, the SR of rats accumulates a lot more Ca^2+^ than what is accumulated during regular stimulation, and the first beat after the rest interval is stronger than the beat before the rest interval. All variables were normalized by the papillary muscle cross-sectional area.

#### Expression of myocardial calcium handling proteins

The following proteins were evaluated by western blot: SERCA2a, PLB, pPLB Ser^16^, pPLB Thr^17^, RYR, CSQ, NCX, and L-type Ca^2+^ channel. Briefly, the LV samples were rapidly frozen in liquid nitrogen and subsequently homogenized in a solution containing RIPA buffer (Amresco LLC, USA) with protease (Sigma-Aldrich, USA) and phosphatase (Roche Diagnostics, USA) inhibitors. The protein samples extracted (50 µg/lane) were subjected to 6–10% SDS-polyacrylamide gel electrophoresis (SDS-PAGE), depending on the molecular weight of the protein. The separated proteins were transferred to nitrocellulose membranes (Armsham Biosciences, USA) and blocked with 5% nonfat dry milk/TBST for 2 h at room temperature. The membranes were incubated overnight at 4°C with primary antibodies against SERCA2a (1:2,500; ABR Affinity BioReagents, USA), PLB (1:1,000, Abcam, USA), pPLB Ser^16^ (1:5,000; Badrilla, UK), pPLB Thr^17^ (1:5,000; Badrilla), RYR (1:5,000; ABR Affinity BioReagents), CSQ (1:300; ABR Affinity BioReagents), NCX (1:1,000; Upstate, USA), and L-type Ca^2+^ channel alpha 1C (1:100; Chemicon International, USA). Then, the membranes were incubated with peroxidase-conjugated secondary antibodies (rabbit or mouse IgG, depending on the primary antibody) for 2 h at room temperature. The blots were detected using Enhanced Chemiluminescence Reagent (ECL, Amersham Biosciences, USA) and analyzed with Scion Image software (Scion Corporation, USA). Targeted bands were normalized to the expression of β-actin (1:1,000; Cell Signaling, USA).

### Statistical analysis

Data are reported as means±SD. Student's *t*-test was used to analyze the results for nutritional profile, comorbidities, cardiac macroscopic and echocardiographic parameters, papillary muscle function at baseline condition, and calcium handling proteins expression. The papillary muscle function after interventions was evaluated using analysis of variance (ANOVA) on the model of repeated measures for independent groups and complemented by the Bonferroni *post hoc* test for multiple comparisons when significant differences were found (P<0.05). The least squares method was used to adjust the linear regression models of DT as a function of the inverse of the length, and of the logarithm of the RT as a function of length for the control and obese groups. The procedure was complemented with statistical comparison of the equality of the models in the two study groups. For all analyses, P*<*0.05 was considered statistically significant.

## Results

### Nutritional profile and comorbidities

Tables 1 and 2 show the nutritional profile and comorbidities of the animals after 33 weeks of treatment. The final body weight, weight gain, naso-anal length, epididymal, retroperitoneal and visceral fat deposits, total body fat, and adiposity index were higher in obese compared to control animals. Although the calorie consumption was similar in both groups and the food intake was lower in the obese animals, the feed efficiency was higher in the Ob group (Table 1).

**Table 1:** Nutritional profile.

	Control (n=18)	Obese (n=19)	P
Initial body weight (g)	167±14	167±15	<0.001
Final body weight (g)	467±52	541±68	<0.001
Naso-anal length (cm)	27.3±0.9	27.8±0.7	0.03
Epididymal fat (g)	7.42±3.03	15.5±4.0	<0.001
Retroperitoneal fat (g)	7.66±3.58	21.8±6.8	<0.001
Visceral fat (g)	5.52±2.16	11.4±3.7	<0.001
Total body fat (g)	20.6±8.2	48.7±13.3	<0.001
Adiposity index (%)	4.52±1.42	9.22±1.54	<0.001
Food intake (g/day)	24.2±2.1	19.8±1.9	<0.001
Calorie intake (kcal/day)	71.3±6.2	72.1±6.9	0.69
Feed efficiency (%)	1.38±0.16	1.72±0.19	<0.001

Data are reported as means±SD. Student's *t*-test for independent samples was used.

Obesity caused significant metabolic and hormonal changes. The systolic blood pressure, AUC, HOMA-IR and triglycerides, total cholesterol, LDL, NEFA, glucose, insulin, and leptin serum levels were significantly higher in the Ob group compared to the C group (Table 2).

**Table 2:** Obesity-related parameters.

	Control (n=18)	Obese (n=19)	P
SBP (mmHg)	121±10	129±67	0.009
Insulin (ng/mL)*	4.46±1.38	6.07±2.27	0.03
Leptin (ng/mL)*	3.58±2.16	16.89±5.90	<0.001
AUC (mg·dL^-1^·min)	18387±3955	24356±4640	<0.001
Homa-IR*	29.3±11.0	42.9±19.0	0.02
Glucose (mg/dL)	104±12	114±12	0.01
Triacylglycerol (mg/dL)	46.7±18.2	72.7±25.4	0.001
Total cholesterol (mg/dL)	68.0±13.4	78.2±14.5	0.04
HDL (mg/dL)	25.1±3.9	27.1±4.7	0.17
LDL (mg/dL)	24.0±4.3	27.7±5.4	0.03
NEFA (mmol/L)*	0.42±0.08	0.51±0.11	0.02

Data are reported as means±SD. Student's *t*-test for independent samples was used. SBP: systolic blood pressure; AUC: area under the curve for glucose; HOMA-IR: homeostasis model assessment of insulin resistance; HDL: high-density lipoprotein; LDL: low-density lipoprotein; NEFA: non-esterified fatty acids. *n=14 for C and n=15 for Ob.

### Postmortem cardiac macroscopic analysis

Tibia length (C: 4.22±0.12 *vs* Ob: 4.30±0.18 cm; P=0.16) and the LV/tibia (C: 0.18±0.02 *vs* Ob: 0.18±0.01 g/cm; P=0.94) and RV/tibia (C: 0.05±0.01 *vs* Ob: 0.05±0.02 g/cm; P=0.90) ratios were not statistically different between the groups. The atrium/tibia ratio (C: 0.019±0.003 *vs* Ob: 0.021±0.004 g/cm; P=0.04) was greater in the Ob compared to the C group.

### Echocardiographic evaluation

Structural and functional echocardiographic data are shown in Table 3. The LVDPWT, LA, and LA/AO were significantly higher in Ob rats; there was no difference in other structural parameters. The systolic function was similar between C and Ob groups, except for PWSV, which was greater in obese rats. The diastolic function parameters had no difference between groups.

**Table 3:** Echocardiographic evaluation.

	Control (n=13)	Obese (n=11)	P
Heart rate (bpm)	261±45	237±23	0.129
LVDD (mm)	7.55±0.54	7.75±0.46	0.328
LVDPWT	1.36±0.05	1.42±0.09	0.036
LVRWT	0.36±0.02	0.37±0.04	0.585
LA (mm)	5.05±0.42	5.47±0.53	0.041
LA/AO	1.28±0.08	1.37±0.11	0.021
FS (%)	51.7±4.2	53.9±5.2	0.254
PWSV (mm/s)	36.7±3.4	41.8±3.8	0.002
E wave (cm/s)	66.8±6.5	71.2±7.1	0.134
A wave (cm/s)	42.0±7.5	43.5±9.0	0.670
E/A	1.63±0.25	1.68±0.25	0.621

Data are reported as means±SD. Student's *t*-test for independent samples was used. LVDD: left ventricle diastolic diameter; LVDPWT: left ventricle diastolic posterior wall thickness; LVRWT: left ventricle relative wall thickness; LA: left atrial diameter; AO: aortic diameter; FS: endocardial fractional shortening; PWSV: posterior wall shortening velocity; E: early diastolic mitral inflow velocity; A: late diastolic mitral inflow velocity.

### Papillary muscle function

**Figure 1:**
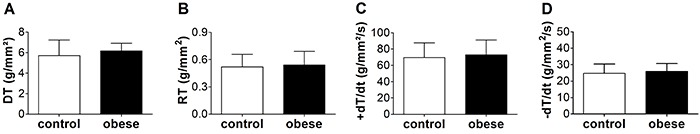
Basal evaluation of myocardial function in papillary muscles from control (n=11) and obese (n=11) rats. **A**, DT: maximum developed tension; **B**, RT: resting tension; **C**, +dT/dt: peak of positive tension derivatives; **D**, –dT/dt: peak of negative tension derivatives. Data are reported as means±SD. Student's *t*-test for independent samples was used to compare data.

**Figure 2:**
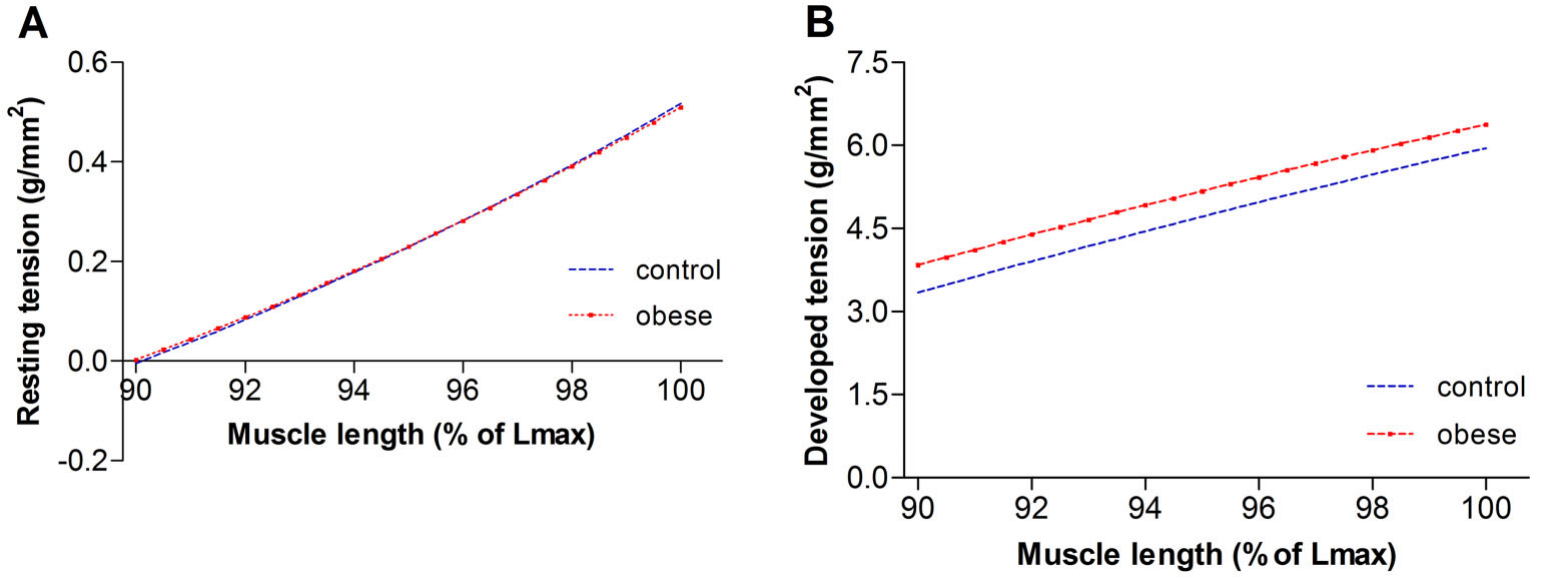
The relationship between muscle length and (**A**) resting tension and (**B**) developed tension from control (n=11) and obese (n=11) rats, using the progression model adjusted according to the group.

**Figure 3:**
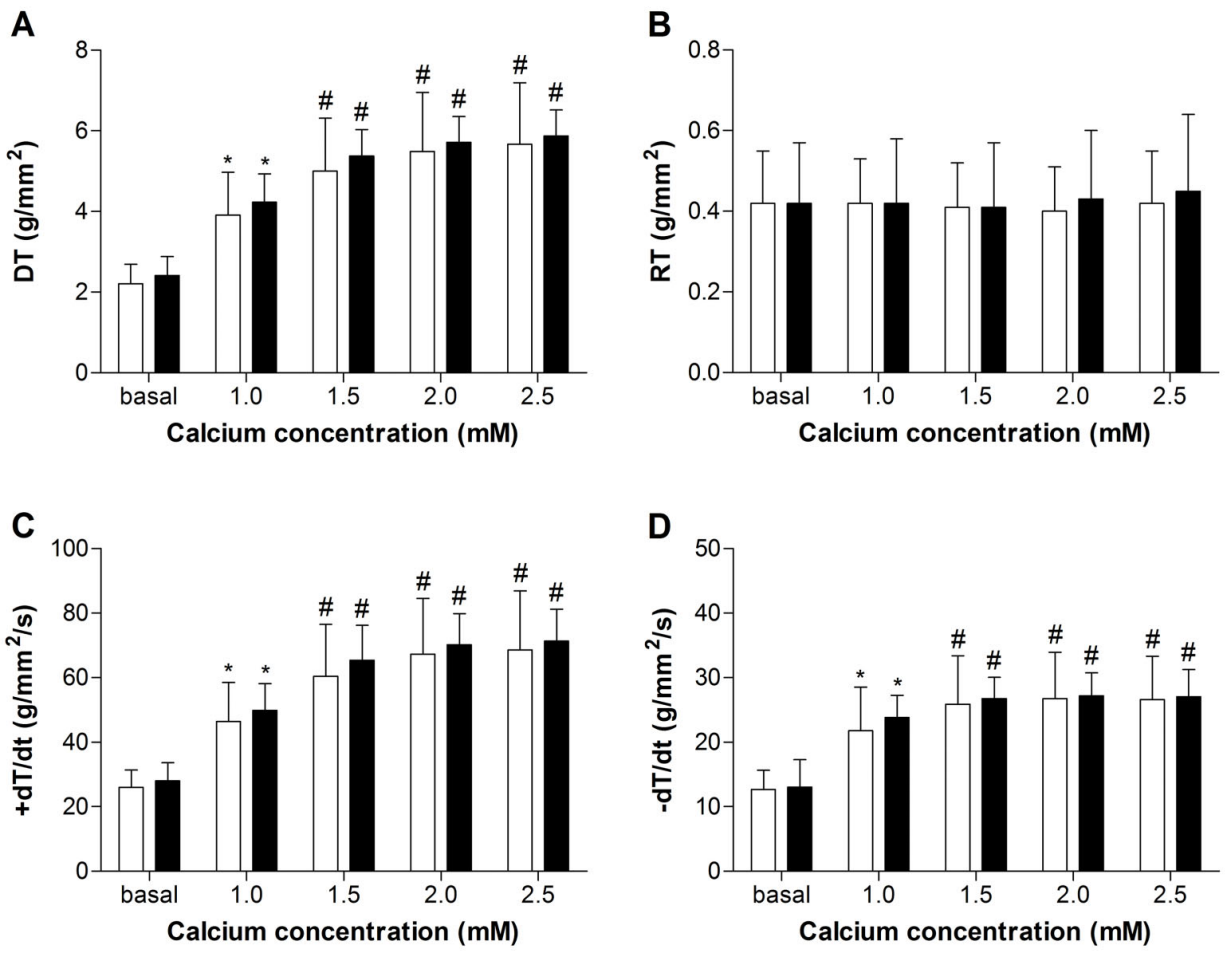
Effects of increased calcium concentration on myocardial function in papillary muscles from control (white bars; n=11) and obese (black bars; n=11) rats after 33 weeks. **A**, DT: maximum developed tension; **B**, RT: resting tension; **C**, +dT/dt: peak of positive tension derivatives; **D**, –dT/dt: peak of negative tension derivatives. Data are reported as means±SD. *P<0.05 *vs* basal; ^#^P<0.05 *vs* basal and 1.0 mM calcium (ANOVA and Bonferroni).

**Figure 4:**
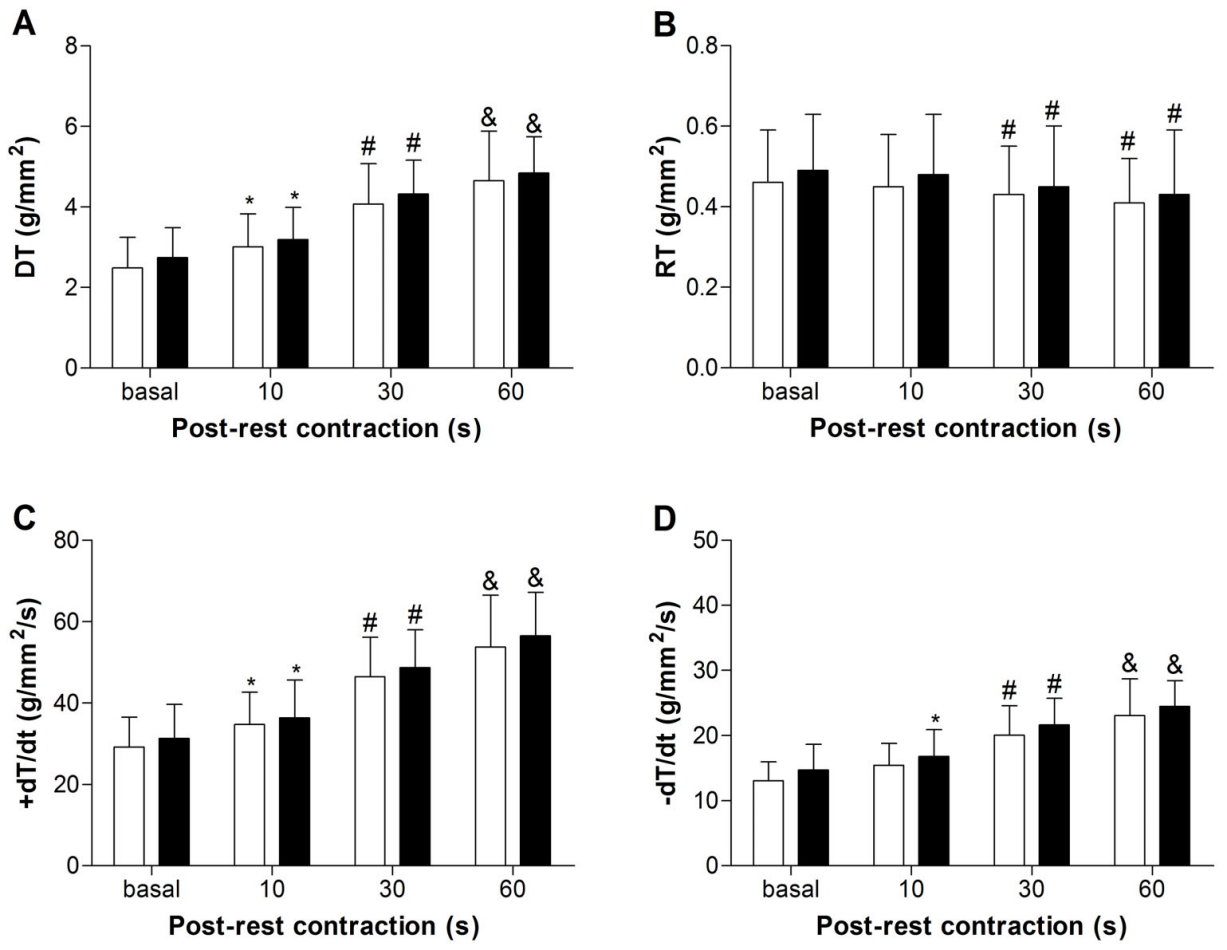
Effects of post-rest contraction on myocardial function in papillary muscles from control (white bars; n=11) and obese (black bars; n=11) rats after 33 weeks. **A**, DT: maximum developed tension; **B**, RT: resting tension; **C**, +dT/dt: peak of positive tension derivatives; **D**, –dT/dt: peak of negative tension derivatives. Data are reported as means±SD. *P<0.05 *vs* basal; ^#^P<0.05 *vs* basal and 10 s; ^&^P<0.05 *vs* basal, 10 and 30 s (ANOVA and Bonferroni).

Figures 1, 2, 3, and 4 summarize the mechanical properties of the isolated papillary muscle of obese and control rats, from the baseline condition and after inotropic intervention. Obesity did not cause contractile dysfunction at baseline condition (Figure 1). The influence of muscular length variation over resting tension and developed tension was similar in both groups (Figure 2). Although the inotropic maneuvers promoted intragroup changes, no differences were observed between groups with increased extracellular Ca^2+^ concentrations and post-rest contraction, suggesting that intracellular Ca^2+^ homeostasis was not compromised in obese rats (Figure 3 and 4).

### Protein expression of myocardial calcium handling

**Figure 5:**
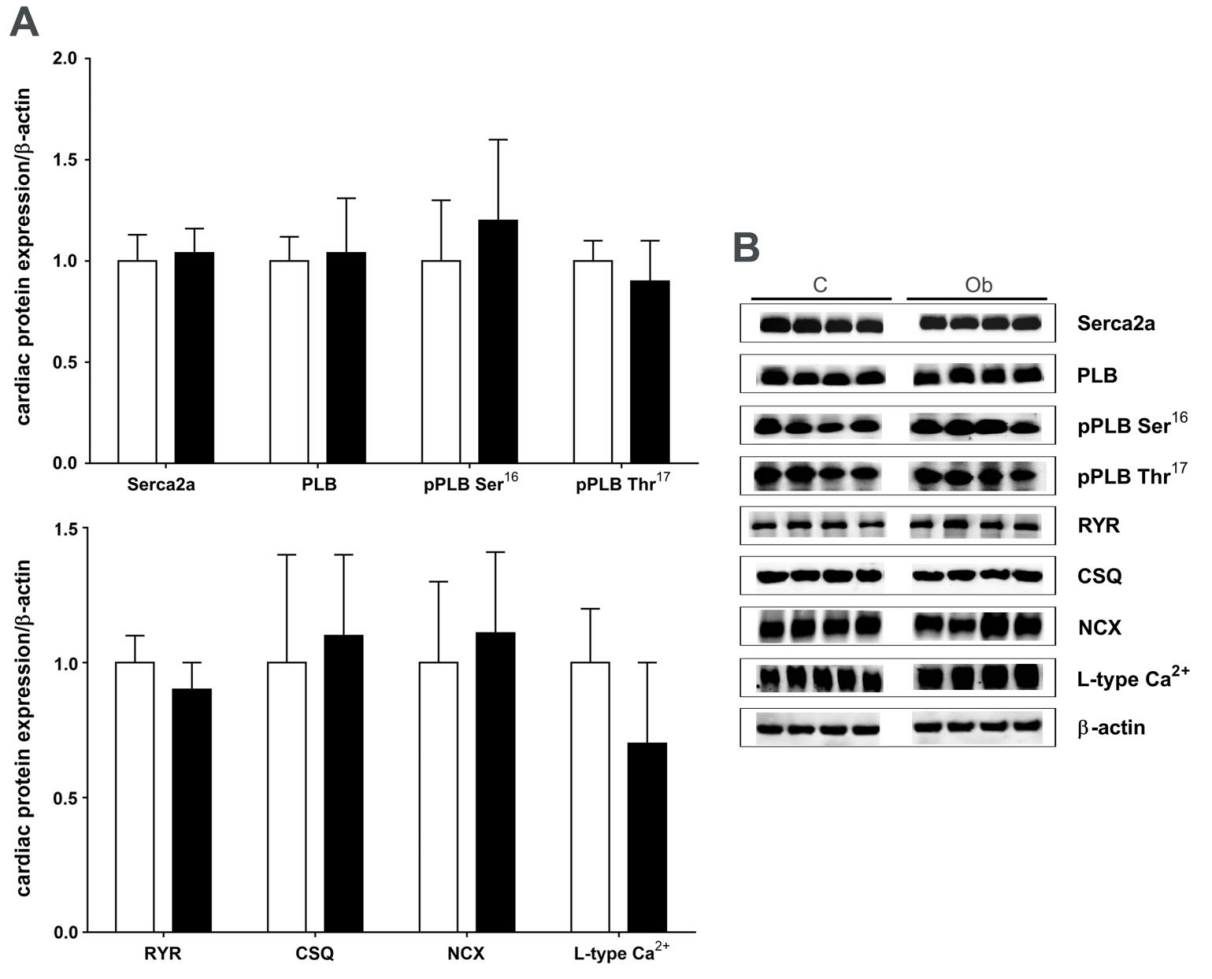
Western blot analysis of intracellular Ca^2+^-cycling proteins in myocardium from control (C, white bars) and obese (Ob, black bars) rats (n=6 in each group). **A**, Quantification of sarcoplasmic reticulum Ca^2+^-ATPase (SERCA2a), phospholamban (PLB), PLB serine-16 phosphorylation (pPLB Ser^16^), PLB threonine-17 phosphorylation (pPLB Thr^17^), ryanodine receptor (RYR), calsequestrin (CSQ), Na^+^/Ca^2+^ exchanger (NCX), and L-type Ca^2+^ channel. **B**, Representative bands of proteins evaluated by western blot. Data are reported as means±SD. Student's *t*-test for independent samples was used to compare data.

**Figure 6:**
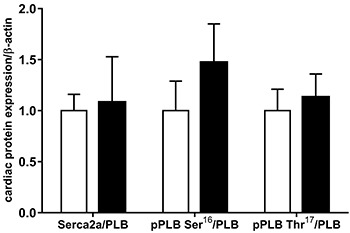
Quantification of sarcoplasmic reticulum Ca^2+^-ATPase (SERCA2a), phospholamban (PLB), serine-16 phosphorylation (pPLB Ser^16^), and PLB threonine-17 phosphorylation (pPLB Thr^17^) normalized to PLB. Control: white bars; Obese: black bars (n=6 in each group). Data are reported as means±SD. Student's *t*-test for independent samples was used to compare data.

Results from intracellular Ca^2+^ cycling proteins SERCA2a, PLB, pPLB Ser^16^, pPLB Thr^17^, RYR, CSQ, NCX, and L-type Ca^2+^ channel are summarized in Figure 5 and 6. Data showed that the expression of these proteins was unchanged by the high-fat diet (Figure 5). Furthermore, the ratios of SERCA2a/PLB, pPLB Ser^16^/PLB, and pPLB Thr^17^/PLB were similar in both groups (Figure 6).

## Discussion

The aim of this study was to investigate whether obesity promotes cardiac dysfunction due to changes in the expression of myocardial regulatory proteins of intracellular calcium homeostasis. The results showed that the excess adipose tissue did not lead to functional cardiac injury nor impaired the proteins related to intracellular Ca^2+^ handling.

Obesity has been extensively studied in experimental models to identify the molecular mechanisms involved in pathological cardiac remodeling. For this purpose, different models with high-fat diets have been used. In this study, a high-fat diet with predominance of saturated fatty acids was used, which was efficient to increase the body weight (16%) and adiposity index (104%) of the rats, corroborating other authors (26,27). These changes occurred despite the lower food intake and similar calorie intake of obese animals compared to controls. According to the literature, saturated fatty acids are usually less oxidized than unsaturated ones; in addition, fat thermogenesis is lower than other nutrients, such as carbohydrates and proteins, thus favoring fat deposition (28–30).

Obese animals presented numerous comorbidities, such as increased blood pressure, glucose intolerance, insulin resistance, dyslipidemia, hyperinsulinemia, and hyperleptinemia, which are found in obese patients with metabolic syndrome (31–33).

Cardiac postmortem morphological analysis showed that these animals presented atrial hypertrophy and did not develop left ventricular remodeling. Structural echocardiographic assessment showed increased left atrial diameter, likely related to hypervolemia or diastolic dysfunction that occur in obesity (34,35).

The proposal of this research was to evaluate the effects of obesity on cardiac function and Ca^2+^ handling-related myocardial proteins. The functional echocardiographic evaluation did not show diastolic dysfunction and revealed change in systolic function by increased PWSV in obese animals, showing that the improvement in myocardial contractility was probably a consequence of adrenergic hyperactivity due to higher leptin, insulin, and fatty acids circulating levels (36,37). The functional study of the isolated papillary muscle detected no difference between the groups at baseline and after inotropic maneuvers that evaluated the calcium participation on myocardium function. This difference in data found between both methods, i.e., echocardiography and papillary muscle, is probably because the heart, *in vivo*, is influenced by the neurohumoral system, which is exacerbated in obesity, which does not occur in the *in vitro* preparation. In addition, the papillary muscle analysis assessed strength parameters, while the echocardiogram assessed the wall movement. One possible factor that may have limited the detection of cardiac dysfunction by echocardiogram in this study can be attributed to the functional evaluation methods of the heart. Perhaps performing the tissue Doppler imaging for myocardial velocities or strain imaging could show additional information. Furthermore, measuring the left ventricular pressure may be helpful to show changes in diastolic function. Therefore, in disagreement with our hypothesis, high-saturated-fat diet-induced obesity did not cause cardiac dysfunction, in agreement with other authors (5,19,27).

Regarding the myocardial proteins analysis related to the intracellular Ca^2+^ handling, obesity did not induce significant changes in the molecules analyzed. Most of the studies found in the literature on this subject used models of obesity induced by high-fat diets rich in unsaturated fatty acids and the results are divergent, probably due to differences in experimental protocols (9,14–18,32). As mentioned previously, Cheng et al. (19), using a high-saturated-fat diet in male Wistar rats for 8 weeks, showed that only pPLB Thr^17^/PLB ratio was downregulated, despite no evidence of left ventricular dysfunction or remodeling. This finding regarding the pPLB Thr^17^/PLB ratio is in disagreement with our results, since none of the protein expressions changed, possibly due to the period of dietary treatment or, more likely, due to the source and percentage of dietary fatty acids. The chosen fat source in the present study was mainly palm kernel oil, which is a vegetable source; therefore, it may contain protective compounds despite being predominantly saturated. Fat from animal sources, like the lard used by Cheng et al. (19), is more often associated with harmful health outcomes (38).

A limitation of this study might be the sample size, since it was not possible to perform the cardiac functional analysis in all animals due to technical problems (e.g., poor image, high heart beat, etc.) in echocardiogram and methodological issues (e.g., similar cross-sectional area between the groups) in the papillary muscle study. This limiting factor might interfere in the power of statistical analysis and consequently in the cardiac function outcomes found in the present study. Despite the fact that the appropriate sample size for basic science research could be a particular challenge, the sample size used in this study was in accordance with several other studies (9,16 [Bibr B17],^19^,^39^,^40^).

In conclusion, our hypothesis was rejected, because obesity induced by the high-saturated-fat diet was not effective in triggering cardiac dysfunction and impairing the proteins related to intracellular Ca^2+^ handling. Further studies in dietary obesity models that result in cardiac dysfunction are required to better clarify the role of the proteins involved in myocardial Ca^2+^ handling on the impairment of heart performance caused by obesity.
